# Analysis of Epstein-Barr virus reservoirs in paired blood and breast cancer primary biopsy specimens by real time PCR

**DOI:** 10.1186/bcr1627

**Published:** 2006-12-12

**Authors:** R Serene Perkins, Katherine Sahm, Cindy Marando, Diana Dickson-Witmer, Gregory R Pahnke, Mark Mitchell, Nicholas J Petrelli, Irving M Berkowitz, Patricia Soteropoulos, Virginie M Aris, Stephen P Dunn, Leslie J Krueger

**Affiliations:** 1 Molecular Genetics, Cellular and Tissue Transplantation, Nemours Biomedical Research, Rockland Road, Wilmington, DE 19803, USA; 2 Department of Surgery of the Alfred I duPont Hospital for Children, Rockland Road, Wilmington, DE 19803, USA; 3 Thomas Jefferson University, Jefferson Medical College, Department of Surgery, Walnut Street, Philadelphia, PA 19107, USA; 4 Oregon Health and Science University, Department of Surgery, Portland, OR 97239-3098, USA; 5 Helen F Graham Cancer Center, Christiana Care Health System, Ogletown Stanton Road, Newark, Delaware 19713, USA; 6 Center for Applied Genomics, Public Health Research Institute, Warren Street, Newark, NJ 07103, USA

## Abstract

**Introduction:**

Epstein-Barr virus (EBV) is present in over 90% of the world's population. This infection is considered benign, even though in limited cases EBV is associated with infectious and neoplastic conditions. Over the past decade, the EBV association with breast cancer has been constantly debated. Adding to this clinical and biological uncertainty, different techniques gave contradictory results for the presence of EBV in breast carcinoma specimens. In this study, minor groove binding (MGB)-TaqMan real time PCR was used to detect the presence of EBV DNA in both peripheral blood and tumor samples of selected patients.

**Methods:**

Peripheral blood and breast carcinoma specimens from 24 patients were collected. DNA was extracted and then amplified by MGB-TaqMan real time PCR.

**Results:**

Of 24 breast tumor specimens, 11 (46%) were positive for EBV DNA. Of these 11 breast tumor specimens, 7 (64%) were also positive for EBV DNA in the peripheral blood, while 4 (36%) were positive for EBV DNA in the tumor, but negative in the blood.

**Conclusion:**

EBV was found at extremely low levels, with a mean of 0.00004 EBV genomes per cell (range 0.00014 to 0.00001 EBV genomes per cell). Furthermore, our finding of the presence of EBV in the tumor specimens coupled to the absence of detection of EBV genomic DNA in the peripheral blood is consistent with the epithelial nature of the virus. Because of the low levels of viral DNA in tumor tissue, further studies are needed to assess the biological input of EBV in breast cancer.

## Introduction

Pathogenic Epstein-Barr virus (EBV) is present in over 90% of the world's population [1,2] and is traditionally associated with relatively benign diseases. However, it is also found in neoplastic diseases, associated with highly aggressive tumor progression and poor patient survival. Endemic Burkitt lymphoma, a subtype of non-Hodgkin lymphoma, is perhaps the best-known example of EBV-associated tumorigenesis. Different models for the emergence of these lymphomas have been hypothesized and some have been verified, although all involve a unique viral-host interaction [3]. Other subtypes of non-Hodgkin and Hodgkin lymphomas are also associated with EBV infections. Perhaps more germane to breast cancer, EBV infection has been associated with other epithelial cancers, for example nasopharyngeal carcinoma and gastric cancer.

In the US, over 200,000 new breast cancer cases are diagnosed per year [4], with approximately 1,000,000 cases diagnosed worldwide [5]. Thus, the association of EBV with breast cancer could profoundly shape clinical diagnosis, disease management and, potentially, patient outcome. As significantly, since EBV is classified as a primary carcinogen in human [6], the potential for EBV carcinogenesis in breast cancer currently concerns the cancer community. Over the past decade, the presence of the virus in breast cancer cells has been controversial. However, when the results were reanalyzed, it was clear that the initial observations were reproducible, verifiable by different laboratories and valid [7]. This conclusion was based on: the overall positive results found in larger studies; the greater sensitivity and specificity of the newer PCR techniques; the attribution of artifacts to the protein reagents used; and the presence of EBV in a subset of other epithelial tumor cells. Other types of problems [7-11] also occurred and were resolved. A major controversy that remains is the interpretation of the negative results obtained when measuring the EBV specific and highly abundant transcripts Epstein-Barr virus encoded small RNAs (EBER I and II) [10]. Unlike the majority of other negative results, this inability to detect EBER transcripts held considerable weight in the diagnostic community. This was attributed to the fact that EBER detection is a standard and has been used diagnostically for a long time. Indeed, it is the method of choice to detect the presence of EBV in putative lymphoma specimens. The answer to this question has been slow to emerge, but a framework is now in place to resolve it [12-14]. One component of the answer is the differential transcription of the genes encoding the EBER transcripts in cells of diverse tissue origins. Another alteration in EBER transcription was documented during lytic cycle expression [12]. These biological concerns, coupled with inherent technical difficulties may, in part, explain some of the discrepancies in measuring EBER.

Since the study of Labrecque and colleagues in 1995 [15], significant efforts have been made to confirm, validate and interpret the EBV findings. With the recent implementation of real time PCR, the emphasis of the initial studies [12,14,16,17] on detecting EBV now includes determination of viral load. Indeed, the emphasis shifted from the percentage of EBV positive specimens to the number of genomes present per cell in those specimens. The remaining question for the future is the biological significance of the low-levels of EBV genomes found. In the current study, quantitative real time PCR (QPCR) was used to detect and quantify the concentration of EBV. The first internal repeat region (IR1) continained in the BamHI W fragment includes from 6 to 12 copies of the repeat as identified in different EBV isolates [18]. The EBV IR1 PCR primers used amplify a 76 base-pair product corresponding to nucleotides 14,649 to 14,722 of the EBV B95-8 strain (NC 001345). We examined both neoplastic breast tissue (*n *= 24) and matched peripheral blood. Comparison of the amounts of EBV DNA in B cell rich peripheral blood with the viral load in the tumor specimens may further help to determine whether EBV is a potentially causal agent or a mere bystander. Using minor groove binding (MGB)-TaqMan probes as opposed to conventional TaqMan probes, we were able to increase the amount of DNA screened per reaction. This facilitated the ability to detect scarce numbers of viral genomes in overwhelming amounts of cellular DNA. Additionally, we determined if there was a correlation between the EBV concentration found in the blood and that found in the biopsy specimens. We propose that the detectable levels of EBV DNA found in the tumor and the absence or lower amounts of viral DNA found in matched peripheral blood supports a relationship between Epstein-Barr virus and breast carcinoma.

## Materials and methods

### Patients and tissue samples

Twenty-four patients with invasive breast carcinoma, meeting the criteria for inclusion as approved by the Institutional Review Board, were enrolled into the study. The demographics of the patient population were collected from the medical records and are summarized in the Results section of this paper. A sequential series of patients were consented and enrolled in the study. A split sample of the specimen from the newly diagnosed carcinoma was taken and examined by a pathologist after hematoxylin-eosin staining. Additionally, the diagnosis of the specimen was determined. Only samples with ≥60% cancer cells were included in the study in accordance with the Internal Review Board approval. Lymphocytes in the tumor were also measured by high-power light microscopy and the counts corrected for volume using the standard correction for microscopic depth of field. We counted 10 fields for each specimen (*n *= 21). Tumor samples were immediately placed in RNA*later*™ (Ambion, Austin, TX, USA) in the operating room. The samples were subsequently stored at -20°C. Fourteen breast cancer lines (MDA-MB231, MDA-MB157, BC3, MDA-MB361, BT20, MDA-MB468, BT474, SKBR3, T47D, MCF-7, MDA-MB-435S, MCF10A, MDA-MB-134-VI and ZR75-1) were included as additional samples.

### Extraction of DNA

DNA was extracted from peripheral blood, cell lines and tissue samples using the QIAamp™ and DNeasy™ Protocols (Qiagen, Valencia, CA, USA). Blood was taken at the time of the initial diagnostic biopsy before instituting therapy. High molecular weight DNA was extracted from the Namalwa cell line (ATCC CRL-1432), breast cancer lines or EDTA-treated blood using QIAamp DNA purification columns (Qiagen) as indicated by the manufacturer. In brief, DNA was isolated from whole blood as follows: a volume of 0.2 ml of blood was treated with 20 units of RNAse ONE (Promega, Madison, WI, USA) at room temperature for 5 minutes. The aliquot was then exposed to protease digestion in the presence of Buffer AL at 56°C for 10 minutes and applied to the column (Qiagen). Each column was sequentially washed with Buffer AW1, Buffer AW2 and finally eluted with 0.2 ml of Buffer AE (Qiagen). Namalwa is a human cell line that contains two copies of the EBV genome in a head to head orientation integrated into the cellular DNA [19]. Namalwa DNA was extracted from 5 × 10^6 ^cells. Calf thymus DNA (Sigma-Aldrich, St. Louis, MO, USA) was purified as described (50 μg/column) and used as a carrier in the construction of the standards for real time PCR. DNA concentrations were determined by comparison to standard curves generated using PicoGreen (Molecular Probes, Eugene, OR, USA) and measured on a Cytofluor Series 400 (Perkin Elmer, Wellesley, MA, USA) at an excitation of 480 nm and an emission of 520 nm.

The tumor tissue was split and added to RNA*later*™ immediately at biopsy. For the tissue, DNA extraction of the initial diagnostic biopsy was performed after the RNA*later*™ fixative was removed by washing the tissue. The tissue was then rehydrated in buffer. This technique can be used to rehydrate protein and DNA, but not RNA. Tumor tissue was obtained from the subjects, handled as described by the manufacturer and diluted in a 10-fold excess of RNA*later*™ and stored at -20°C. Samples were stored in RNA*later*™ for subsequent studies requiring stabilized RNA. For the isolation of DNA from extracted tissue, 30 mg of individually cut and pulverized tissue samples were placed in 1.5 ml microcentrifuge tubes and washed twice with normal saline to remove the RNA*later*™. The samples then had 180 μl of buffer ATL added, followed by 20 μL of proteinase K, and were incubated at 55°C overnight. This was performed to improve tissue lysis in the samples that were lysis-resistant. Then, 4 μl of RNAse A were added to the samples, which were incubated at 70°C for 10 minutes after adding 200 μl of buffer AL. We then added 230 μl of ethanol (modification of the protocol suggested by the manufacturer to optimize viral nucleic acid recovery), followed by transfer of the samples to the DNeasy mini columns. Buffer AW1 (500 μl) was added and the samples centrifuged at 6000 × g for 1 minute. This was followed by the addition of 500 μl of buffer AW2 and centrifugation at full speed for 3 minutes. A 200 μl aliquot of buffer AE was added to the DNeasy membrane, followed by incubation at room temperature for 1 minute and centrifugation at 6000 × g for 1 minute. DNA was concentrated using Pellet Paint (Novagen, San Diego, CA, USA) as per the instructions of the manufacturer. The concentrated pellets were air-dried overnight and resuspended in 40 μl of distilled water. Sample DNA was quantified using PicoGreen DNA dye (Molecular Probes) as described by the manufacturer. The PicoGreen reagent was prepared in a 1:200 dilution (50 μl of PicoGreen per 10 ml 1× TE Buffer). The PicoGreen reagent was then added in 100 μl aliquots to each well and mixed. The plate was read with the CytoFluor system (excitation 485/20, emission 530/25, 3 reads/well, 1 cycle, 4 scans/cycle, gain 50; Turner Biosystems (Sunnyvale, CA, USA); the R-value was found to be 0.9983.

### Amplification of DNA using MGB-TaqMan PCR

DNA amplification was accomplished by real time monitoring of fluorescence intensity during PCR using MGB-TaqMan probes. We prepared 1.1 μg of DNA per reaction and reactions were run in triplicate for samples, standards and controls (with the exception of two tumors for which insufficient tumor sample was available). Amplification was performed in a total of 20 μl containing 2× Universal TaqMan PCR master mix (Applied Biosystems, Foster City, CA, USA) 900 nM of BAMHIW (forward primer, 5'-CCC AAC ACT CCA CCA CAC C-3'; reverse primers, 5'-TCT TAG GAG CTG TCC GAG GG-3'), 250 nM of Probe LIR-1MGB2 (FAM-ACACTACACACACCCACC-MGBNFQ) and 1.75 μl of water. Namalwa DNA [19] was used to prepare the standards (using 20, 200, 2,000, and 20,000 EBV copies per reaction; calculated using 2 EBV genomes per 7 pg of Namalwa DNA), and Daudi cells were used as an additional positive control. The 7900 Sequence Detection System program (Applied Biosystems) used was 95°C for 10 minutes followed by 40 cycles of 95°C for 15 seconds, 60°C for 1 minute. Fluorescent measurements were obtained throughout the amplification process.

### Statistics

Real time PCR was performed and the absolute quantity of each amplicon was calculated using the algorithm provided in SDS version 2.1 as described in User Bulletin #2, (Applied Biosystems). Calculations of the mean polymerase chain cycle number at which the intensity of the amplicon was greater than 10× the standard deviation of the threshold (CT-value), standard deviation and mean EBV genomes per reaction were performed using SDS version 2.1. A two-sided *t*-test was performed for the lymphocytes present in the blood [20] and quantified in the tumor specimens (see above). Additional statistical analyses were calculated using SPSS software version 11 (Chicago, IL, USA).

## Results

### Patient population

Of the 24 breast carcinoma specimens studied, demographic data were obtainable for 23 female patients (Table 1). The age distribution was: <45 years, 3 (13%); 45 to 55 years, 8 (35%); and >55 years, 12 (52%). Tumor size ranged from <2 cm, (52%) to ≥2 cm, (48%). With regard to axillary nodal status, 11 (48%) did not have nodal metastasis; 2 (9%) had micrometastasis to the axillary lymph nodes and 9 (39%) had positive lymph nodes. There were 16 (70%) pre-menopausal patients and the remaining ones were post-menopausal. There were 6 (27%) estrogen receptor positive specimens, while 16 (73%) were negative and one undetermined; and 8 (36%) were progesterone receptor (PR) positive with 14 (64%) negative and 1 specimen unknown. Twenty (95%) patients were positive for the Her2/Neu oncoprotein, with the results from two patients undetermined. Concerning histology, specimens were classified using the WHO classifications. We found that 16 (80%) patients had an infiltrating mixed carcinoma (mixed ductal and lobular carcinoma or infiltrating carcinoma with mixed ductal and lobular features or mixed ductal and lobular differentiation). One patient (5%) had a ductal carcinoma *in situ*, one (5%) had an infiltrating mixed carcinoma; while an additional patient (5%) had a mucinous and one (5%) had an infiltrating lobular carcinoma; results for four patients were unknown. The distribution of tumor stage was as follows: stage I, 9 (45%); stage IIA, 5 (25%); stage IIB, 4 (20%); and stage IIIA, 2 (10%); and the results for four patients were unknown. Patients with stage IIIB or stage IV tumors were not seen and, therefore, patients with these stages were not enrolled in the study.

### EBV load measurements

**Figure 1:**
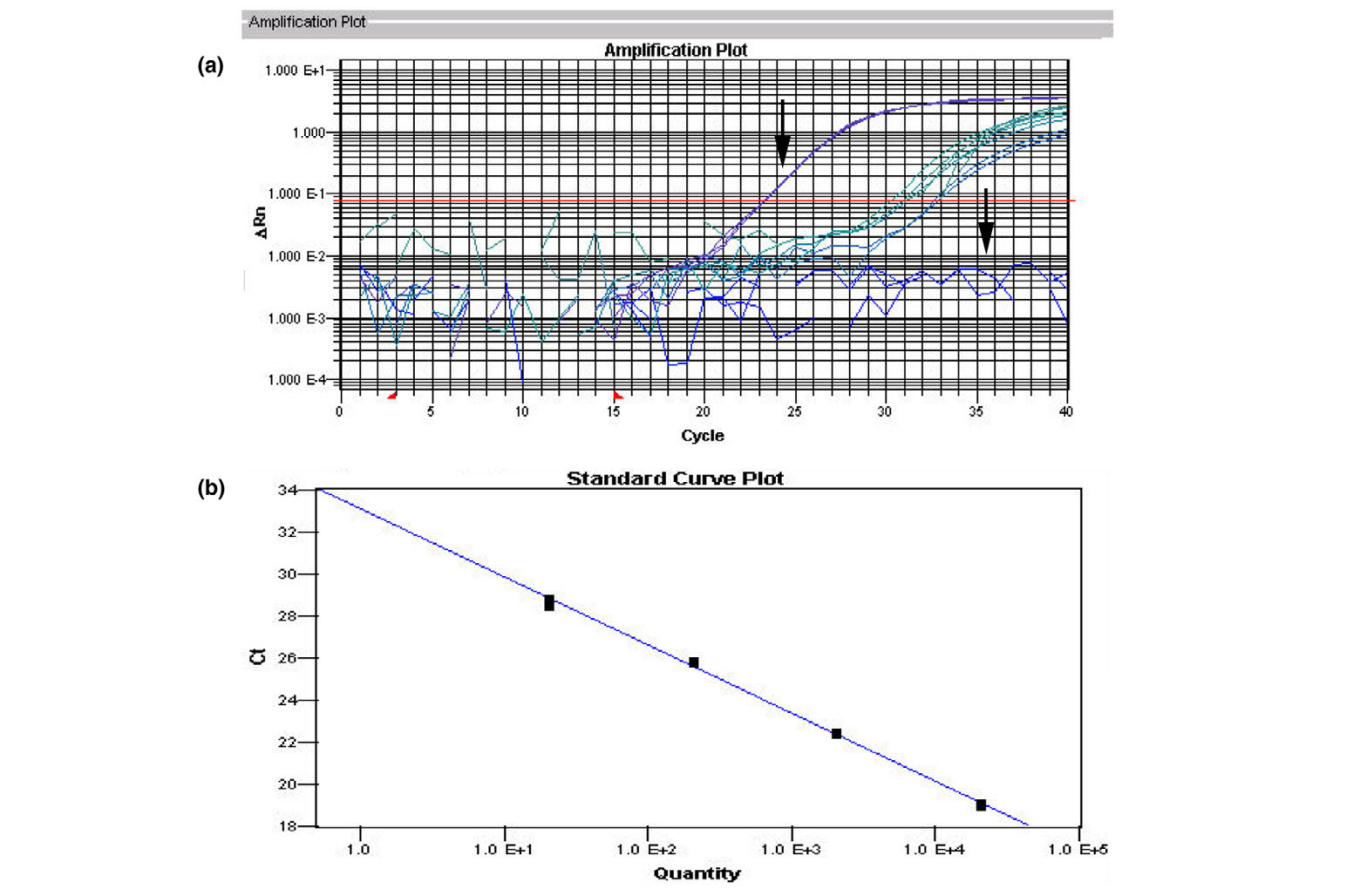
Amplification of Epstein-Barr virus (EBV) from DNA isolated from breast cancer and blood specimens. Real time PCR using minor groove binding (MGB)-TaqMan technology was used to quantify the viral load contained in the samples. Internal repeat region (IR)1 target sequences showed that this probe amplified its respective target over a broad range and detected low levels (2.3 EBV genomes per reaction; unpublished observation). **(a) **A characteristic amplification plot showing the change in fluorescence (ΔRn) as a function of amplification cycle. The horizontal red line indicates the fluorescence at 10× the standard deviation of the control. The upper left arrow indicates the fluorescence detected from Daudi, an EBV-associated endemic Burkitt lymphoma. The lower arrow indicates the fluorescence of a negative control (water). The amplification, in triplicate, of the DNA from each of the patient tumor samples is indicated. **(b) **The standard was constructed to contain from 2 to 200,000 copies of EBV genome. The graph shows the linear regression of the Cts (the PCR cycle number when the amplification fluorescence value reaches and exceeds the predetermined background threshold value) using each of the standards. This characteristic standard line had an r^2 ^= 0.995 with a slope of -3.2.

Real time PCR using MGB-TaqMan technology was used to determine whether DNA isolated from blood and breast cancer specimens contained EBV DNA (Figure 1a). To quantify the amount of EBV DNA in the unknown specimens, EBV standards were prepared using Namalwa DNA (two copies of EBV per cell) in the presence of 1,100 ng of total calf thymus carrier DNA and the appropriate amount of Namalwa DNA. This assured that the complexity of the experimental determinations, the total DNA concentration in the reactions, were similar to that of the standards. The standards were linear over several magnitudes of changes in the EBV concentration (Figure 1b). In the amplification study, we used a ribosomal DNA probe/primer set (Applied Biosystems) to identify either the total absence of amplification or inhibition of the PCR reaction caused by the quality of the extracted DNA. Of the 24 breast tumor specimens, 11 (46%) were positive for EBV DNA. Of these 11, 7 (64%) were also EBV positive in the peripheral blood and the other 4 negative positive tumor samples were negative in the paired blood specimens. Conversely, 3 of 10 (30%) patients were positive for EBV DNA in the peripheral blood but this was undetected in the tumor. Overall, 10 of the 24 (42%) patients' peripheral blood specimens were positive for EBV DNA while 11 (46%) were positive for EBV in the tumor DNA. These results include seven patients that were positive in both the blood and the tumor (Table 2). Using as the diploid standard 7 pg of DNA per human genome, cell equivalence was calculated in the blood and tumor DNA. No correction was made for the potential variation in aneuploidy/polyploidy of the tumor population. The mean concentrations of EBV in these positive patient specimens were 8.0 per 160,000 cell equivalents of DNA isolated from peripheral blood. In the tumor, 5.0 EBV genomes per 160,000 cell equivalents of DNA were documented. No demographic trends were observed but there were too few cases for formal statistical analysis. Additionally, none of the 14 cell lines used as controls was positive for EBV DNA.

## Discussion

A current concern of the cancer community has been the potential of viral mediated cancer [2]. Over the past decade, the detection of EBV in a high percentage of breast cancer patients has driven efforts to verify the presence of the virus in epithelial cells and their tumors, to quantify the amount of virus present and to identify the role that EBV might be playing in the initiation and dissemination of breast cancer as well as other solid tumors. Previous reviews have focused on the technical difficulties and disparities obtained when different approaches for measuring EBV [9,12,21,22] were used. In addition, extensive studies of breast cancer cells [23] and this report show that EBV viral DNA was undetectable in these established cell lines. After reviewing the previous studies, we found that five showed consistently negative results. Those studies used immunohistochemistry or *in situ *hybridization to detect EBER l, EBER ll, EBNA-2, or EBV latent membrane protein-1 (LMP-1) [24-28]. In contrast, most studies showing greater than 50% positivity for EBV employed PCR [13,29-31]. In addition, the two largest studies showed the presence of EBV in 162 out of 509 and 51 out of 100 specimens [29,31]. Only 1 of 12 studies that used real time PCR had a negative result for the presence of EBV DNA. In that study [8], EBV was measured after performing LCM on the tumor samples; the authors had originally found 21% positivity by QPCR and interpreted the laser capture microscopy (LCM) results to suggest that EBV could not be derived from tumor cells since, after LCM, none of the samples was positive for EBV DNA [8]. This interpretation might be limited, for example, if only a very small subset of cells within the tumor was EBV positive. The dissection itself, therefore, would significantly limit the ability to detect an EBV positive cell. On the other hand, two other studies performed QPCR of tumor samples and showed that the homogenous breast cancer cells were heterogenous with respect to the number of EBV genomes detected per cell and also showed cells containing multiple EBV genomes [31,32]. These results ultimately led to the general acceptance that breast cancer cells harbor EBV. Thus, the detection of EBV in breast cancer biopsies supports a potential role for this human carcinogen in this malignancy.

The role of EBV in breast neoplasms is also supported by the ability of the virus to infect epithelial cells and to synthesize epithelial-specific viral transcripts [12,33-37]. Various research groups were able to infect established human transformed epithelial cell lines, as well as primary epithelial cells [12,33-38]. It is now apparent that infection occurs more readily through cell-to-cell co-culture with infected B cell lines [34-36] than by free-viral particle infection [12,38]. In addition, it was demonstrated that the lower efficiency of direct viral infection could be compensated using G418 antibiotic selection [12]. The susceptibility of these cells to EBV was initially surprising because the cells lacked the classic B cell CD21 EBV receptor. The absence of this receptor suggests the presence of a different cell mediated uptake mechanism for EBV. Although CD21-mediated viral uptake is seen in some epithelial cells, EBV infection in these cells is mediated by the gp25-gp85 complex [39-42]. Taken together, these studies suggest that EBV infects epithelial cells through a mechanism different from B-cells.

Lastly, the finding of a unique set of viral transcripts in epithelial cells reinforces the support for the role of EBV in breast carcinoma. This set, while overlapping several B-cell transcripts, differs from those found in B cells in major ways. For example, Xue and colleagues [13] evaluated 15 breast cancer specimens obtained by mastectomy and showed that the 2 small viral RNAs, EBER I and II, could not be detected. This result strongly suggested that breast cancers showed an altered pattern of EBV transcripts. Recently, Arbach and colleagues [32] confirmed the previous results [13] and showed that EBNA-1, an early transcript derived from an open reading frame encoding a 26- to 33-kilodalton protein that is recognized by anti-EA serum (BARF-1), and LMP-1 transcripts were present in 77%, 57% and 20% of the breast cancer cases analyzed, respectively. Significantly, these results also underscored the potential mechanism for the negative EBER staining previously shown in other breast cancer studies. Heretofore, EBER staining was the gold standard for the diagnosis of EBV infection. At present, it is clear that application of this diagnostic should be considered for cell types showing classic EBER positivity, for example, B cell, T cell and T/NK cell lymphomas, nasopharyngeal carcinoma, and gastric cancer. Additionally, seven EBV transcripts found in B-cells (EBER l and ll, EBNA-1 transcribed from either the Cp or Wp promoters, the *Bam*HI rightward reading frame 1), a viral member of the Bcl-2 family of apoptosis-regulating proteins (BHRF-1), LMP-1 and LMP-2 were negative in the breast cancer biopsies. In addition, the promoter for a family of alternatively spliced *Bam*HI-A rightward transcripts CST (BARTs) and the IR4/F3 (leftwardreading frame 3) a replication-associated transcript containing the IR4 (PstI restriction enzyme repeats) are usually found in carcinomas. The DNA sequence of the IR4 transcript from a nasopharyngeal carcinoma tumor, C15, shows sequence variation that is different from those reported for B cells or from an established Burkitt cell line (Raji). Additionally, EBNA1 transcripts from the Qp promoter were found in the majority of breast cancer biopsies positive for EBV [13]. Huang and colleagues [12] extended the study described above through the use of an *in vitro *model system. The results of this study clearly demonstrated a transcript profile in the infected breast cancer cells characteristic of EBV-induced latency II. In a small percentage of cells, a transcriptional pattern associated with active lytic infection was also found. These results were further extended to ten snap frozen samples of late stage invasive breast carcinoma biopsies and confirmed the presence of lytic infection. The positive findings showed that the EBV lytic population was not specific to their model system, but also occurred *in vivo *in breast cancer.

We were able to detect EBV in freshly obtained breast cancer biopsies using QPCR. Our data confirm previous studies using both endpoint and real time PCR analyses. We show that a high percentage of breast cancer biopsies (46%) were EBV positive. Unexpectedly, we found that the EBV positive tumor biopsies contained an extremely low viral load. If each genome represents an infected cell, the average incidence of infection in the EBV-associated population was 0.00004 (ranging from 0.00014 to 0.00001) in blood. In the breast tumor biopsies positive for EBV, the average was 0.00002 EBV genomes per cell (ranging from 0.00009 to 0.00001). This confirms a recently reported similarly low EBV load [8,12,14,16]. This is especially significant in that the samples used here and by Huang and colleagues [12] were fresh biopsies. In contrast, the results described by the study of Ryan and colleagues [14] used archival material extracted from paraffin-embedded tissues. Additionally, the heterogeneity of EBV genomes per cell must be considered. Here, we found very low levels of EBV genomes per cell and Murray and colleagues [8] found levels intermediate to these, while the EBV genomes per epithelial cell found by Arbach and colleagues [32] overlapped the studies. Arbach and colleagues measured EBV genome levels in EBV positive breast cancer specimens (*n *= 44) and found that EBV DNA was present in 23% of them at a level of ≤0.1 genomes per 1,000 cells, in 45% at a level of ≤1.0 genomes per 1,000 cells and in 32% at a level of >1.0 genome per 1,000 cells.

Another aspect of this study [32] investigated the likelihood of inflammatory, virally infected B cells contaminating the breast cancer biopsy and, therefore, being responsible for the presence of the EBV viral DNA. Previous investigators proposed that bystander lymphocytes might account for the measured percentage of EBV positive breast cancers [13,24,43,44]. Huang and colleagues [12] demonstrated that the levels of EBV were similar between tumor epithelial specimens and control specimens. In their study, however, the control material was taken from the same tissue and may not be considered normal. To further investigate the bystander phenomenon, we used real time PCR to show that the EBV load in the blood of these breast cancer patients was also extremely low (3 genomes ± 5 per 1.57 × 10^5 ^cells per reaction, 42% of specimens positive). These low blood titers make it extremely unlikely that the 11 tumors were positive as a result of a passive bystander effect (Table 2), especially considering the increased lymphocyte count in the blood versus the tumor (p ≤ 0.05). Additionally, positive EBV levels in the tumor material with undetectable titers in the blood also support this hypothesis. In the same manner, tumors with viral titers greater in the tumor than in the blood reinforce the previous observations. Taken together, our data continue to support the epithelial nature of the virus found. Additionally, the carcinogenic impact of a limited EBV infection remains relevant if one considers either 'hit-and-run' infection [45], or stem cell infection [46,47]. In the case of the stem cell, differentiation of the infected stem cell could developmentally kick the virus out, thereby leaving an uninfected cancer. Determination of the impact of a limited EBV infection on breast epithelial cells awaits future research.

## Conclusion

Our data support that EBV infection, albeit at low viral titer per cell, is present in approximately half of all breast cancer biopsies.

## Abbreviations

EBV = Epstein-Barr virus; IR = internal repeat region; LCM = laser capture microscopy; LMP = latent membrane protein; MGB = minor groove binding; QPCR = quantitative real time PCR.

## Competing interests

The authors declare that they have no competing interests.

## Authors' contributions

RSP, KS, NJP, IMB, SPD and LJK were involved in all aspects of this work, including the planning stages and its performance. They were also involved in discussions and interpretation of the data, as well as manuscript preparation. PS and VMA were involved in aspects of the breast cancer cell work and interpretation, discussion and manuscript preparation. CM was responsible for the MGB-TaqMan experiments and the formulation of the data for manuscript presentation. MM was responsible for the pathological evaluation and staining of the appropriate slides for interpretation. MM was involved in discussions on the lack of correlation and its meaning with respect to the demographic data. DD-W and GRP were involved in the original design and IRB proposal for this work. They continued their efforts throughout the course of this work.

## Figures and Tables

**Table 1:** Demographics of the breast cancer population

Variable	Number
Age, years (*N *= 23)	
<45	3
45–55	8
>55	12
Menopausal status (*N *= 23)	
Pre-/peri-menopausal	16
Post-menopausal	7
Tumor size (cm) (*N *= 23)	
<2	12
≥2	11
Nodal status (*N *= 22)	
Negative	11
Positive	11 (2 micrometasis)
Unknown	1
Tumor grade (SBR) (*N *= 20)	
I	9
II	9
III	2
Histology (*N *= 20)	
Ductal	16
Lobular	1
Other	3
ER status (*N *= 22)	
Positive	6
Negative	16
Unknown	1
PR status (*N *= 22)	
Positive	8
Negative	14
Unknown	1
HER-2 status (*N *= 21)	
Positive	20
Negative	1
Unknown	2

*N *= the number of specimens available for evaluation. ER, estrogen receptor; PR, progesterone receptor; SBR, Scarff, Blum, and Richardson.

**Table 2:** Analysis of the concentration of EBV in matched blood and tumor specimens

Category	Peripheral blood-/tumor-	Peripheral blood+/tumor+	Peripheral blood+/tumor-	Peripheral blood-/tumor+
Number (percentage of total)^a^	10 (42)	7 (29)	3 (12)	4 (17)
Ratio of EBV genomes^b ^in blood versus tumor (range of EBV levels in peripheral blood)	0.0	5.0 (0.6–22.0)	Undefined	Undefined
Range of EBV levels^c ^in peripheral blood	0.0	8.0 (2.0–22.0)	4 (1.0–8.0)	0.0
Range of EBV levels^c ^in tumor specimens	0.0	5.0 (1–14)	0.0	1.0 (1.0–2.0)

^a^*N *= 24. ^b^The number of Epstein-Barr virus (EBV) genomes was determined in 160,000 cell equivalents of DNA isolated from peripheral blood and tumor specimens. ^c^Range (highest level to lowest level of EBV genomes in the peripheral blood).
